# Thermal Stability of Cpl-7 Endolysin from the *Streptococcus pneumoniae* Bacteriophage Cp-7; Cell Wall-Targeting of Its CW_7 Motifs

**DOI:** 10.1371/journal.pone.0046654

**Published:** 2012-10-08

**Authors:** Noemí Bustamante, Palma Rico-Lastres, Ernesto García, Pedro García, Margarita Menéndez

**Affiliations:** 1 Instituto de Química-Física Rocasolano, Consejo Superior de Investigaciones Científicas, and Centro de Investigación Biomédica en Red de Enfermedades Respiratorias (CIBERES), Madrid, Spain; 2 Centro de Investigaciones Biológicas, Consejo Superior de Investigaciones Científicas, and Centro de Investigación Biomédica en Red de Enfermedades Respiratorias (CIBERES), Madrid, Spain; University of South Florida College of Medicine, United States of America

## Abstract

Endolysins comprise a novel class of selective antibacterials refractory to develop resistances. The Cpl-7 endolysin, encoded by the *Streptococcus pneumoniae* bacteriophage Cp-7, consists of a catalytic module (CM) with muramidase activity and a cell wall-binding module (CWBM) made of three fully conserved CW_7 repeats essential for activity. Firstly identified in the Cpl-7 endolysin, CW_7 motifs are also present in a great variety of cell wall hydrolases encoded, among others, by human and live-stock pathogens. However, the nature of CW_7 receptors on the bacterial envelope remains unknown. In the present study, the structural stability of Cpl-7 and the target recognized by CW_7 repeats, relevant for exploitation of Cpl-7 as antimicrobial, have been analyzed, and transitions from the CM and the CWBM assigned, using circular dichroism and differential scanning calorimetry. Cpl-7 stability is maximum around 6.0–6.5, near the optimal pH for activity. Above pH 8.0 the CM becomes extremely unstable, probably due to deprotonation of the N-terminal amino-group, whereas the CWBM is rather insensitive to pH variation and its structural stabilization by GlcNAc-MurNAc-l-Ala-d-isoGln points to the cell wall muropeptide as the cell wall target recognized by the CW_7 repeats. Denaturation data also revealed that Cpl-7 is organized into two essentially independent folding units, which will facilitate the recombination of the CM and the CWBM with other catalytic domains and/or cell wall-binding motifs to yield new tailored chimeric lysins with higher bactericidal activities or new pathogen specificities.

## Introduction

Bacteriophages and bacteria produce a variety of peptidoglycan-degrading enzymes, the cell wall hydrolases (CWHs) [1], either to lyse host cells (endolysins) or to re-model the cell wall during growth and division (endogenous CWHs). Because of their potent ability to digest the cell wall of Gram-positive bacteria when exogenously added, endolysins represent a novel class of antimicrobials. They provide a mean of selective and rapid killing of pathogenic bacteria with no effect on the normal microbiota [1], [2]. Endolysins also exhibit low toxicity, moderate inhibition by the host immune response and a low probability of developing resistance [3]–[5]. These unique features have boosted the interest on the biotechnological and pharmacological exploitation of endolysins, including prevention and treatment of drug-resistant infections, decontamination and food biopreservation [1], [6]–[8].

Endolysins are frequently composed of one or more catalytic units linked to a number of other modules commonly involved in cell wall attachment [2], [6], [8]. In contrast to the current advance of research on endolysins' bacteriolytic activity, the knowledge regarding endolysin receptors on the bacterial envelope is relatively scanty. Cpl-7, the endolysin encoded by the *Streptococcus pneumoniae* bacteriophage Cp-7, consists of an N-terminal catalytic module (CM) with muramidase (lysozyme) activity fused to a C-terminal module containing 3 identical tandem repeats (hereafter called CW_7 repeats) of 42 amino acid residues each [9]. The CW_7 repeats are essential for the activity [10] and unique among the sequences of the CWHs encoded by the pneumococcus and its bacteriophages [11]. They confer to the Cpl-7 endolysin the ability to degrade pneumococcal cell walls containing either choline or ethanolamine and to retain full activity in the presence of high choline concentrations. In contrast, all other pneumococcal CWHs behave as choline-dependent enzymes since their cell wall-binding modules recognize the choline moieties present in pneumococcal (lipo)teichoic acids [12]. The gain-of-function on ethanolamine-containing cell walls shown by the chimeric LC7 amidase, built by fusion of the CW_7 repeats of Cpl-7 to the CM of pneumococcal LytA amidase, provided further evidence for the involvement of the CW_7 repeats on cell wall targeting [13]. However, the nature of the receptors on the bacterial envelope remains unknown. A three-dimensional model of the Cpl-7 endolysin has been recently proposed [14]. According to it, the CM and the cell wall-binding module (CWBM) form an elongated particle of ≈115 Å long where the CM, folded into the (βα)_5_β_3_ barrel characteristic of the GH25 family [15], laterally packs through the last three β-strands to the superhelix-like structure formed by the three CW_7 repeats, each of them folded into a three-helical bundle.

Although the CW_7 repeats were firstly identified as a component of the Cpl-7 endolysin [9], similar motifs have been subsequently observed, fused to a variety of catalytic units and cell wall-binding modules, in many proteins putatively involved in cell wall metabolism that are encoded by Gram-positive and Gram-negative bacteria as well as by bacteriophages infecting Gram-positive bacteria [14]. In this respect, the ability of CW_7 motifs to target a structural element of the bacterial envelope common to several species, some of them well-recognized pathogens, offers interesting perspectives for exploiting CW_7-containing lysins as enzybiotics with broader spectra of lytic activities or for pathogen detection [8], [16]. Of note, acquisition of CW_7-like motifs by the endopeptidase module of the λSa2 endolysin, a bifunctional enzyme encoded by the LambdaSa2 prophage of *Streptococcus agalactiae*, not only increased its activity on several streptococcal strains when externally added, but was essential for degradation of staphylococci [16].

Structural stability and binding specificity are relevant features for the pharmacological or biotechnological use of proteins. This paper reports thermal denaturation studies of the Cpl-7 endolysin aimed to examine its structural stability under a wide range of pHs and to identify the receptor of the CW_7 motifs on the bacterial surface, using both circular dichroism (CD) and differential scanning calorimetry (DSC).

## Materials and Methods

### Proteins and chemicals

Cpl-7 lysozyme was purified from extracts of *Escherichia coli* RB791 [pCP700] cells, as previously described [14]. Mass spectrometry determination of Cpl-7 molecular mass yielded 38,107 Da, in agreement with the theoretical value estimated from the sequence reported in the database (Entrez code, AAA72844) after processing of the N-terminal methionine (38,116.8 Da). The fragment of Cpl-7 comprising the CM was obtained by controlled proteolysis with trypsin, using a Cpl-7/protease ratio (w/w) of 22 (3 h incubation in 0.1 M phosphate (Pi) buffer, pH 7.0) [10]. The proteolytic fragments were loaded into a DEAE-cellulose column (1.5×3 cm) equilibrated with the same buffer and the CM was eluted with a linear NaCl gradient (0–0.5 M in 40 min). The purity and length of the isolated CM was checked by SDS-PAGE and MALDI-TOF MS. Mass data were consistent with fragments comprising residues 2–203 and 4–203 (data not shown). Before use, proteins were extensively dialyzed at 4°C against the appropriate buffer. Protein concentrations were measured spectrophotometrically using molar absorption coefficients of 71,700 M^−1^ cm^−1^ and 42,930 M^−1^ cm^−1^ for the full length protein and the CM, respectively. Net charges of Cpl-7 and its structural elements were estimated with the program Sendterp [17].

Chitin oligomers [(GlcNAc)_2_, (GlcNAc)_4_ and (GlcNAc)_6_] were provided by Toronto Research Chemicals, MurNAc-l-Ala-d-isoGln and GlcNAc-MurNAc-l-Ala-d-isoGln muropeptides by Sigma and Calbiochem, respectively, and trypsin by Promega. All other reagents (analytical grade) were from Sigma.

### Mass spectrometry

Protein samples were analyzed by MALDI-TOF as described elsewhere [18]. A grid voltage of 93%, a 0.1% ion guide wire voltage, and a delay time of 350 ns in the linear positive ion mode were used. External calibration was performed with carbonic anhydrase (29,024 Da) and enolase (46,672 Da) from Sigma, covering an *m/z* range from 16,000 to 50,000 units.

### Enzymatic activity assays

Cpl-7 enzymatic activity was measured at 37°C in the pH interval comprised between 4.4 and 7.5 using [*methyl*-^3^H]choline-labeled pneumococcal cell walls as substrate [13]. Measurements were carried out in 20 mM Pi buffer (pH 6–7.5) or 25 mM sodium acetate (pH 4.4–6.0) and the proportionality between the amount of cell walls solubilized and the reaction time was verified at all pHs.

### Circular dichroism

CD spectra were recorded using a Jasco-810 spectropolarimeter equipped with a Peltier-thermostatted cell holder. Measurements were performed at different pHs using protein concentrations of 0.2 mg ml^−1^ (1 mm path-length cells) and 1 mg ml^−1^ (10 mm path-length cells) in the far- and near-UV regions, respectively. Collected spectra were the average of three-to-four accumulations and the raw data were converted to molar ellipticities after subtraction of the buffer contribution using, as indicated, either the average molecular mass per residue (111.6 and 113.3 Da for Cpl-7 and CM-Cpl-7, respectively) or the molecular mass of the molecule. Typically, thermal denaturation was monitored by measuring the ellipticity changes at 195, 203, 208 and 290 nm as the temperature was raised at 20 or 40°C h^−1^. Experiments run in the presence of peptidoglycan analogues were followed only at 290 nm because of the absorption in the far-UV region at the employed concentrations. In selected series, sample heating was paused at defined temperatures to allow complete CD spectra to be recorded. All the denaturation profiles obtained at a given pH were analyzed simultaneously in terms of multiple, two-state independent transitions using equation [1]:
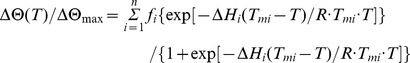
(1)where ΔΘ(*T*) is the ellipticity change at temperature *T* (Kelvin degrees) at the selected wavelength, ΔΘ_max_ is the final variation in the ellipticity at that wavelength, *R* is the gas constant, *f_i_* is the relative contribution of transition *i* to ΔΘ_max_, and *T_mi_* and Δ*H_i_* are the half transition temperature and the enthalpy change for transition *i*, respectively [19]. The non-lineal fitting of experimental profiles was performed using the Origin software (Microcal, Inc) imposing, at each pH, the same values of *T_mi_* and Δ*H_i_* for a given transition at all the wavelengths.

Ligand binding affinity of GlcNAc-MurNAc-l-Ala-d-Iso-Gln was estimated from the increase of the CWBM stability induced by the ligand addition using equation [2]:
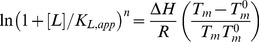
(2)where it is assumed that the heat capacity increment for CWBM denaturation was negligible [20]. 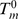 and *T_m_* are the half transition temperatures of the CWBM in the absence and in the presence of the ligand at concentration [L], respectively, Δ*H* is the denaturation enthalpy, *K_L_* is the ligand dissociation constant at 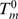, and *n* is the number of ligand molecules bound to the CWBM in the complex.

### Differential scanning calorimetry (DSC)

DSC measurements were carried out using MCS-DSC or VP-DSC microcalorimeters (Microcal, Inc.) in the buffers used for CD studies. Cpl-7 samples (0.4–1 mg ml^−1^) and buffers were degassed before loading into the calorimeter and the scans were run under an extra constant pressure of 2×10^5^ Pa. Standard MCS- and VP-Viewer softwares were used for data acquisition, and Origin-DSC for data analysis. The excess heat capacity functions were obtained by dividing the raw data by the number of protein moles in the DSC cell, after subtraction of the buffer-buffer base line. The reversibility of thermal transitions was checked by reheating samples after a first scan, and runs at several heating rates were performed to examine the potential influence of kinetic effects on the heat capacity profiles.

## Results

### Cpl-7 thermal stability: pH dependence

Conformational stability is an absolute requirement for the pharmaceutical use of proteins with folded native structures. To this aim, thermal denaturation studies can provide an accurate evaluation of conditions that minimize unfolding and maintain full biological activity. They also permit protein ligands to be identified since complex formation stabilizes protein structures. Moreover, in modular proteins whose modules unfold separately, denaturation studies can provide a way to identify the regions involved in specific ligand recognition if the protein transitions have been previously assigned [20]. DSC and CD spectroscopy were therefore used to investigate the thermal stability of Cpl-7 endolysin and its dependence on pH, and also to search for the receptor of CW_7 motifs on the bacterial envelope.

#### CD analysis

**Figure 1:**
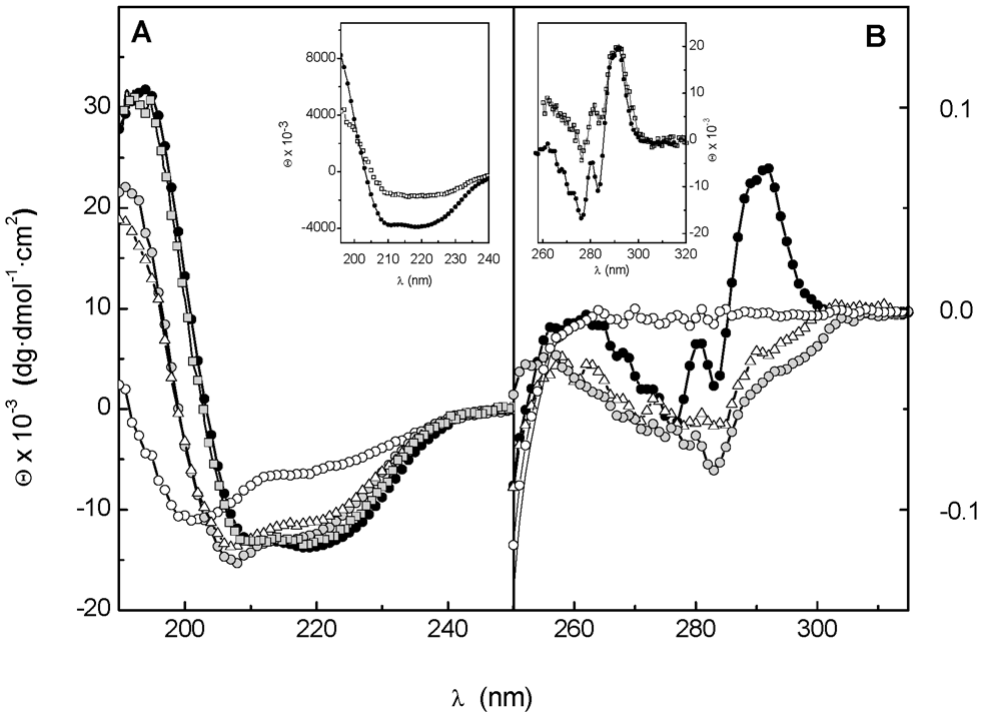
CD spectra of the full-length Cpl-7 endolysin and its isolated catalytic (CM) module. (**A**) and (**B**) show the far- and near-UV CD spectra, respectively, of the full-length protein at 20°C (black circles), 55°C (triangles), 95°C (white circles). Spectra obtained under renaturing conditions (20°C) with protein samples heated up to 90°C (grey circles) and 55°C (grey squares) are also depicted. The insets compare the spectra of CPL-7 (black circles) and the isolated CM (white squares) with the ellipticities expressed per mole of molecule.

**Figure 2:**
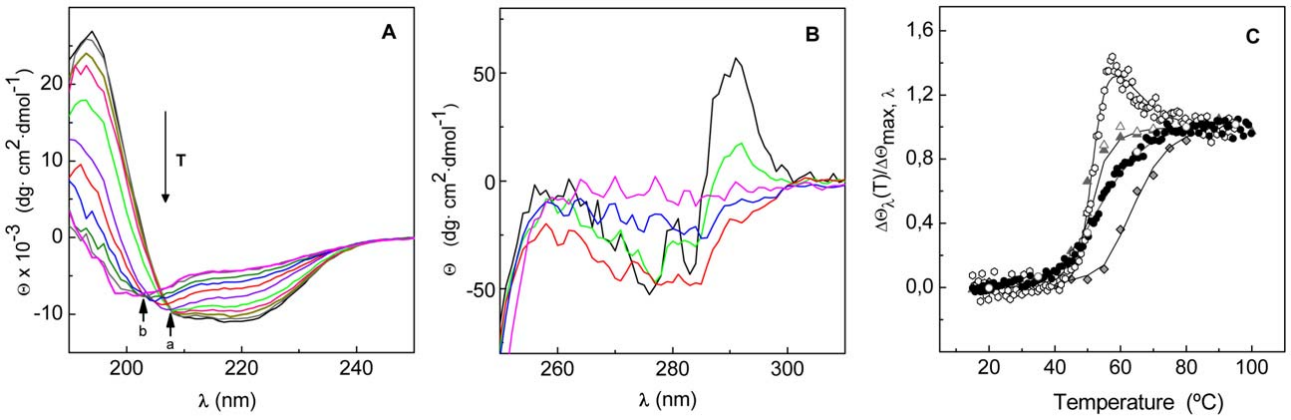
Dependence of Cpl-7 CD spectra with temperature at neutral pH. (**A**) and (**B**) show the spectroscopic changes induced by the temperature increase in the far- and near-UV regions, respectively. Isodichroic points in the far-UV region are marked as “a” and “b”, and the T arrow indicates the temperature increase (20, 30, 40, 45, 50, 55, 60, 65, 70, 85 and 95°C). The near-UV spectra correspond to 20°C (black), 50°C (green), 55°C (blue), 65°C (red) and 95°C (magenta). (**C**) compares the thermal denaturation profiles of Cpl-7 at 195 nm (circles), 203 nm (triangles), 208 nm (diamonds) and 290 nm (hexagons). Measurements were performed in 20 mM Pi buffer, pH 7.0, at heating rates of 20°C h^−1^ (solid symbols) and 40°C h^−1^ (open symbols).

Figure 1 shows the far- and near-UV CD spectra of the Cpl-7 endolysin. Under native conditions, the far-UV CD spectrum shows the typical features of proteins with high α-helical content (Figure 1A), supporting the recently proposed three-dimensional model of the protein [14]. In the near-UV region (Figure 1B) the spectrum shows a well defined positive band centered at 292.6 nm, due to tryptophan residues, together with several negative bands attributable to the fine structure of aromatic residues [21]. The two spectra remained essentially unchanged in the whole range of pH tested (from 5.5 to 8.5) although some loss of native structure was observed at room temperature in the experiments performed at pH 8.5 (see below). The spectroscopic complexity of Cpl-7 denaturation is illustrated in Figure 2. At neutral pH, denaturation begins around 35°C and the spectra collected in the far-UV region show an isodichroic point at around 208 nm that moves to 203 nm as the temperature goes over 50°C (Figure 2A). The trend was similar in the near-UV region where the loss of the positive band centered at 292.6 nm occurred between 35°C and 55°C (Figure 2B), while the negative peaks at lower wavelengths disappeared at higher temperatures (55°C to 80°C). Denaturation was therefore monitored by following the ellipticity *vs* temperature in the proximity of the isodichroic points (203 and 208 nm) as well as at 195 nm (a maximum in α-helix spectrum) and 290 nm. Visual inspection of the denaturation profiles revealed that changes at 203 and 208 nm seem to correlate, primarily, with the low- and high-temperature processes monitored at 290 nm, respectively (Figure 2C). In contrast, variations at 195 nm cover the full protein denaturation range (Figure 2C). On the other hand, the reversibility experiments showed a partial recovery of the native features when the protein was heating up to 90°C (Figure 1), and the spectra monitored under renaturing conditions were similar to those registered at around 55°C during the protein heating. However, when the thermal scan was stopped at intermediates temperatures (∼55°C), the spectra recovery after Cpl-7 cooling to 15°C exceeded 97% (Figure 1A). Besides, variation of the heating rate had only minor effects on the shape and position of the thermal profiles. These observations suggest that i) irreversibility primarily affects to the regions of Cpl-7 unfolding at lower temperatures, and ii) the irreversibly denatured form/s are scarcely populated within the temperature-interval where the respective transition/s take place. In these conditions, the analysis of Cpl-7 denaturation using equilibrium thermodynamics would not lead to significant errors [22], since the ratio between the native and denatured forms involved in each transition will be close to that of the equilibrium species within the transition interval.

**Figure 3:**
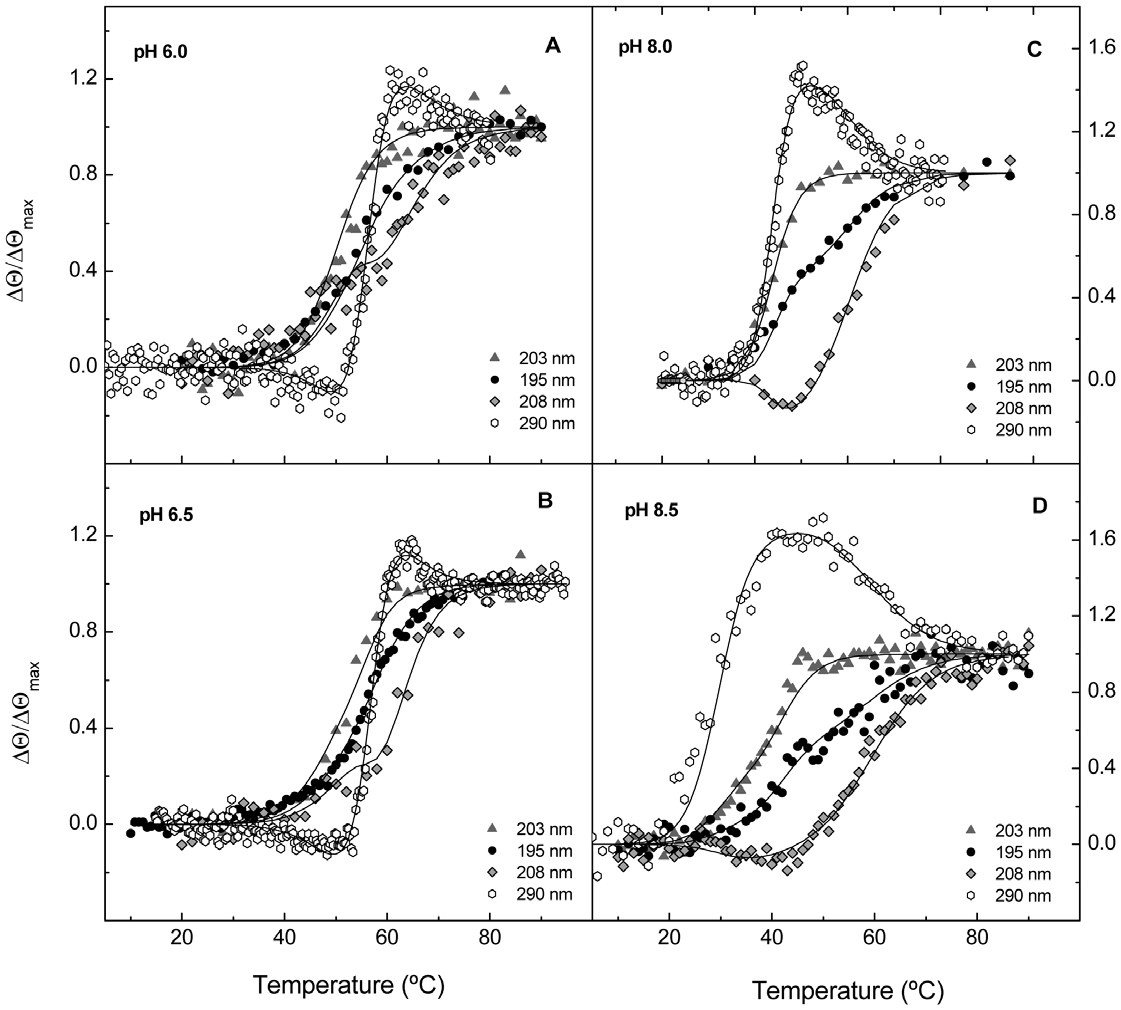
Influence of pH on Cpl-7 thermal stability. Panels (**A**) to (**D**) depict full-length protein denaturation profiles at 195 nm (circles), 203 nm (triangles), 208 nm (diamonds) and 290 nm (hexagons) at different pHs. Continuous lines are the theoretical curves calculated as indicated in the text using the fitting parameters shown in Table 2. Measurements were carried out in 20 mM Pi buffer (pH 6.0, 6.5 and 8.0) and 25 mM sodium borate buffer (pH 8.5) at a scan rate of 20°C h^−1^ (195, 203 and 208 nm) and 40°C h^−1^ (290 nm).

The inspection of the denaturation profiles collected at different pHs showed that Cpl-7 overall stability is maximal around pH 6.0–6.5, slightly above the optimum pH for activity (Figure S1), though the distinct transitions are differently affected by the solvent acidity (Figure 3). Above neutral pH, the decay of the 290-nm positive-band present in Cpl-7 spectrum as well as the variations at 203 nm are drastically shifted to lower temperatures. Besides a rather moderate increase in the negative ellipticity at 208 nm seems to precede the decrease observed at higher temperatures (Figure 3C and D). As inferred from these observations, the conformational stability of the Cpl-7 regions accounting for the respective process/es are dramatically compromised above neutrality, becoming marginal at around 20°C at pH 8.5 (Figure 3D). In contrast, the increase in the medium acidity primarily affects the shapes of the 208-nm and 290-nm thermal profiles (Figure 3A and B); the former shows an inflection around 60°C, and a third phase became visible below 50°C in the latter, suggesting that the loss of the negative component of the 290-nm band might proceed in two steps under acidic conditions (Figure S2).

**Figure 4:**
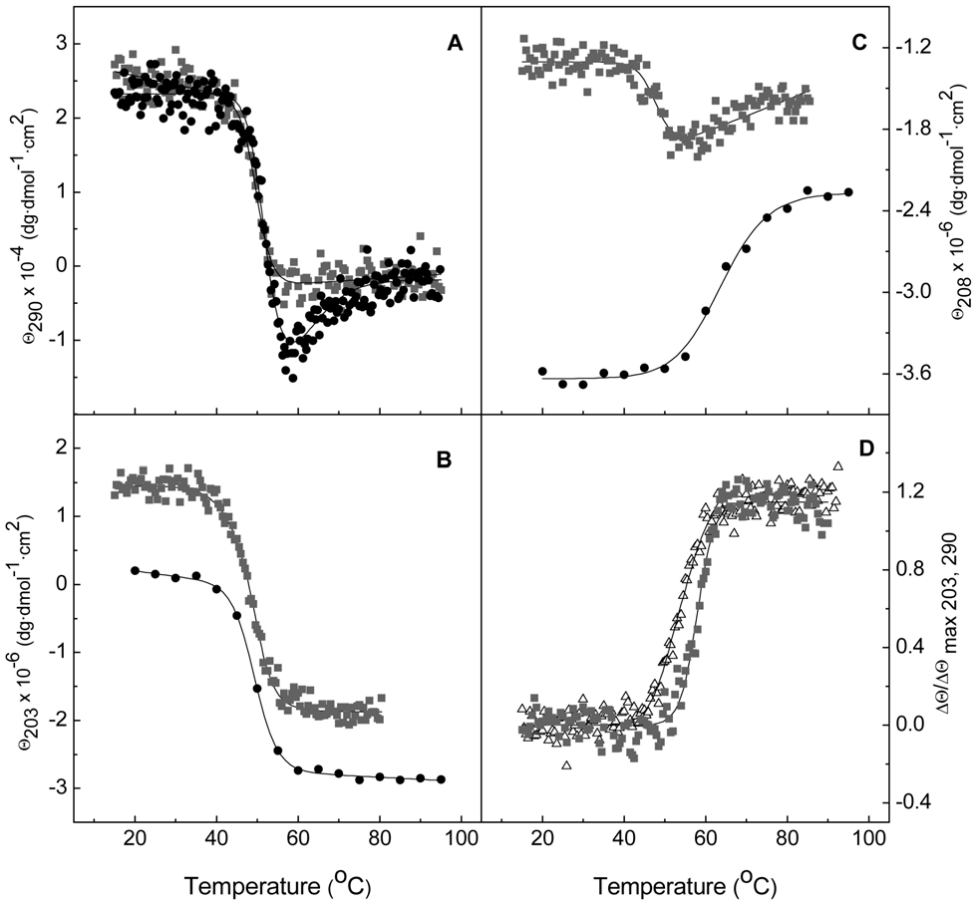
Comparison of the full-length protein and the CM thermal transitions. (**A**), (**B**) and (**C**) compare the CD denaturation profiles of Cpl-7 (black circles) and the CM alone (greysquares) monitored at 290 nm, 203 nm and 208 nm, respectively, in 20 mM Pi buffer pH 7.0 (scan rate of 40°C h^−1^). For comparison, ellipticities per mole of molecule were used instead of the average molar ellipticities per residue. (**D**) shows the differences in the relative ellipticity *vs* temperature profiles at 290 nm (squares) and 203 nm (triangles) for the CM denaturation at pH 6.0. The continuous traces are the curves calculated as indicated in the text with the best fitting parameters (Tables 1 and 2).

To assess whether the CD transitions could correlate with the denaturation of Cpl-7 modules, a truncated form of the endolysin comprising the CM alone was obtained by controlled proteolytic digestion with trypsin [10], as detailed in Materials and Methods. The near-UV CD spectrum of the CM showed the strong positive peak at 292 nm previously observed in the full-length protein spectrum (Figure 1B), but essentially lacked the negative components observed below 286 nm. Interestingly, when the molar protein concentration (instead of the molar average residue concentration) was used for raw data normalization, the spectra of the CM and the full-length protein almost superimpose above 285 nm. Besides, at 290 nm, the CM denaturation profile and the first phase observed in the curve of the full-length protein fully superimpose (Figure 4A), evidencing that it was due to the CM denaturation; the second step will therefore correspond to denaturation of the CWBM. The 203-nm denaturation profiles of Cpl-7 and the CM also display similar trends (Figure 4B) although, at this wavelength, the absolute ellipticities differ due to the strong contribution of the CWBM to the full-length protein far-UV spectrum [14]. On the contrary, both modules contribute to the ellipticity changes observed at 208 nm (Figure 4C) and 195 nm (data not shown).

As shown in Figure 4D, the CM denaturation profiles at 203 nm and 290 nm differ at pH 6.0. This observation indicates that the CM unfolding may proceed in two steps whose transition temperatures approach as the pH was increased, in spite of the high cooperativity usually displayed by α/β barrels and other supersecondary structures. At around pH 8.0, the two steps either overlap or merge in a single process, although, according to the Cpl-7 denaturation at pH 8.5, they separate again at higher pH (Figure 3D). Moreover, the inversion in the order in which ellipticity variations at 203 nm and 290 nm begin as the pH was increased (Figure 3) strongly indicates that they might arise from denaturation of distinct structural elements of the CM rather than from stabilization of an intermediate in the unfolding pathway. Interestingly, thermal denaturation of the isolated catalytic domain of Cpl-1 endolysin, which shares 85.6% sequence with the CM of Cpl-7, takes place at similar temperatures and might also proceed in more than one step at pH 7.0, considering the asymmetry of its DSC profile and the ratio between the calorimetric and van't Hoff denaturation enthalpies [23]. Table 1 summarizes the apparent thermodynamic parameters derived from fitting of CM denaturation in terms of one or two two-state independent processes (see the Materials and Methods), depending on the pH. According to them, only one of the two transitions seen during CM denaturation at neutral and acidic pH contributes to the ellipticity changes at 290 nm, which should be due to variations in the environment of tryptophan side-chains.

**Table 1:** Dependence on pH of CM-Cpl-7 thermal denaturation parameters.

pH	*T_mi_*	Δ*H_i_*	*f_i_* (θ_203_)	*f_i_* (θ_290_)
	(°C)	(kcal/mol)		
6.0	51.4	76	0.72	0.001
	58.5	94	0.28	0.999
7.0	46.2	53	0.49	0.001
	49.8	108	0.51	0.999
8.0	43.8	76	1	—
	43.5	98	—	1

*T_mi_* and Δ*H_i_* are the half transition temperature and the enthalpy change for transition *i*, respectively and *f_i_* is the relative contributions of transition *i* to the total ellipticity change at a given wavelength. Incertitude in fitted parameters are ≈0.2–1% for *T_mi_*, 3–15% for Δ*H_i_* and ±(0.1–0.2) for *f_i_*.

Taken together, CD denaturation data strongly indicated that the loss of the native structure of Cpl-7 might proceed in several steps. The whole set of denaturation profiles obtained at every pH for the full-length protein was therefore simultaneously analyzed in terms of multiple, independent two-state transitions using equation (1) and selecting the simplest model able to provide a good description of the experimental curves. As shown in Table 2 and Figure 4, Cpl-7 denaturation can be described in terms of three apparently independent two-state transitions, with the exception of pH 8.0 where similar chi-square deviations between experimental and theoretical data were obtained assuming only two two-state transitions. The relative contribution of each transition to the ellipticity change at the different wavelengths supported the notion that CM denaturation is responsible for the two lower-temperature transitions, whereas the CWBM would primarily account for the last, pH-insensitive step. Nevertheless, at acidic pH, a small implication of the CWBM in the first transition cannot be fully discarded, considering the estimated contributions of this module to the ellipticity changes at 208 nm and 290 nm when denaturation takes place at neutral or basic pHs. Inclusion of a fourth transition at pH 6.5 and below to separate the potential contribution of the CM and the CWBM to this first step did not improve the goodness of the fits, and the errors of the fitting parameters were significantly higher. On the other hand, the analysis of CD profiles assuming that ellipticity changes arising from the CWBM denaturation take place in a single step at all pHs yielded values of *T_m_*_3_ significantly lower than the respective DSC estimations at acidic pH (see below). It is worth noting the good correlation found between the denaturation parameters estimated for the CM transitions by fitting the thermal profiles of the module alone or within the full-length protein (Tables 1 and 2). On the other hand, the rather small contribution of the CM region responsible for the ellipticity variations at 290 nm to the 195-nm thermal profile suggests that it should primarily comprise *β*-strands. Indeed, three of the four CM tryptophans are located in the *α*/*β*-barrel side devoid of α-helices (Figure S3).

**Table 2:** Denaturation parameters of Cpl-7 endolysin derived from CD thermal profiles at different pH values.

pH	*T_mi_* (°C)	Δ*H_i_* (kcal/mol)	*f_i_* (θ_195_)	*f_i_* (θ_203_)	*f_i_* (θ_208_)	*f_i_* (θ_290_)
5.5	48.0	36	ND	0.6	0.3	−0.2
	52.5	103	ND	0.4	−0.1	1.8
	59.9	48	ND	—	0.8	−0.6
6.0	50.2	53	0.4	0.8	0.5	−0.3
	56.3	100	0.2	0.2	−0.3	1.8
	62.2	41	0.4	—	0.8	−0.5
6.5	49.6	45	0.5	0.7	0.3	−0.3
	57.0	101	0.1	0.3	−0.3	1.8
	61.5	59	0.4	—	1.0	−0.5
7.0	50.9	40	0.7	0.5	—	—
	51.7	101	—	0.5	−0.1	1.6
	62.0	46	0.3	—	1.1	−0.6
8.0	43.9	93	0.5	1	−0.2	1.7
	60.8	46	0.5	—	1.2	−0.7
8.5	42.5	61	0.4	0.6	—	—
	29.4	58	0.1	0.4	−0.1	1.7
	59.9	38	0.5	—	1.1	−0.7

*T_mi_*, Δ*H_i_* and *f_i_* are defined as in Table 1. The uncertainty in the fitting parameters was 0.2–1% in *T_mi_*, 3–15% in Δ*H*_i_, and ±(0.1–0.2) in *f_i_*. ND, not determined.

#### DSC analyses

**Figure 5:**
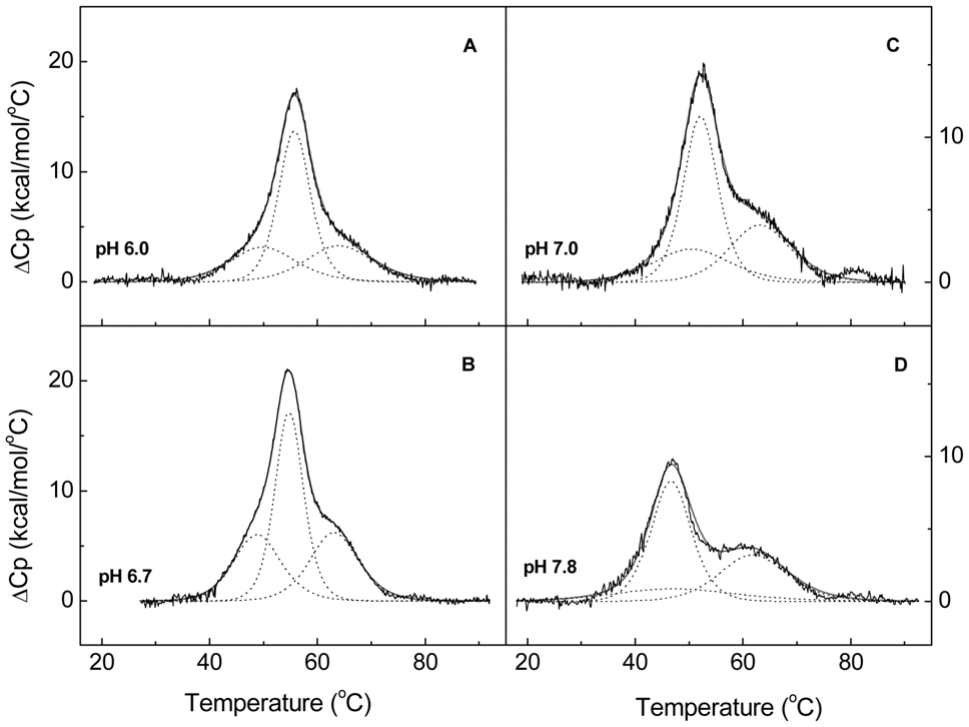
Influence of pH on Cpl-7 DSC denaturation profiles. Panels (**A**) to (**D**) show the thermograms registered at 20°C h^−1^ in 20 mM Pi buffer at the pH values indicated in the curve labels. Dotted lines depict the results of endotherm deconvolution in independent two-state transitions (Table 3) and grey traces are the theoretical envelopes.

**Figure 6:**
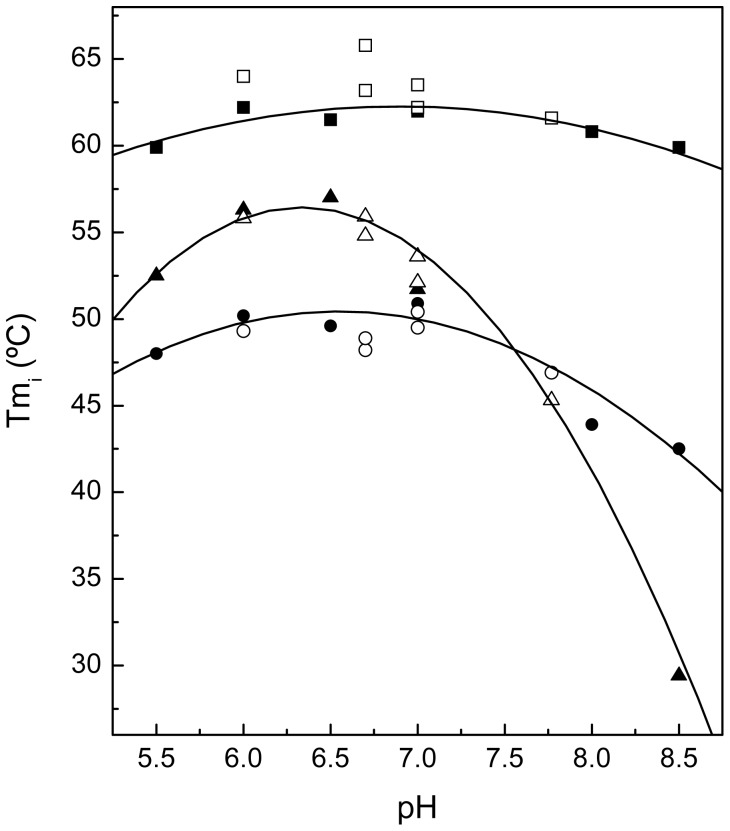
Dependence of *T_mi_* values of Cpl-7 transitions with pH. Solid and open symbols represent the estimates from CD and DSC denaturation profiles, respectively: *Tm*_1_, circles; *Tm*_2_, triangles; and *Tm_3_*, squares.

Cpl-7 stability was further assessed by DSC. Figure 5 shows the heat capacity curves of the full-length protein at different pH values. Denaturation proceeds with a total enthalpy change of 220±20 kcal mol^−1^ at pH 6.0 where the thermogram exhibits a wide peak centered at 55.8°C and two shoulders at around 46.7°C and 65.4°C. As the pH increases, the main peak shifts towards the low-temperature shoulder and both transitions move to lower temperatures (Figure 5A–D). As in CD experiments, the thermograms monitored at 40°C h^−1^ and 20°C h^−1^ were similar, and the last transition was still visible when a second scan of the sample was run (data not shown). Deconvolution of DSC curves showed that, in the range of assayed conditions, Cpl-7 denaturation can be described in terms of three two-state apparently independent transitions, which further supports the previous analysis of CD profiles. The thermodynamic denaturation parameters derived from the DSC experiments are shown in Table 3. It is worth noting the good correlation existing between them and the transition parameters derived by CD analyses (Figure 6). Moreover, the deconvolution of the DSC curve recorded at pH 7.8 strongly indicate that the two well-resolved CM transitions observed at acidic pHs tend to merge in a single process as their *T_m_*'s approach each other at around pH 8.0 (Figure 5D). Indeed, the transition parameters derived at pH 7.8 for the main peak (*T_m2_* = 46.4°C and Δ*H_2_* = 82 kcal mol^−1^) are close to those estimated by CD for the CM either alone (*T_m_* = 43.6°C and Δ*H* = 87 kcal mol^−1^; average of 203-nm and 290-nm fits) or in the full-length protein (*T_m1_* = 43.9°C and Δ*H_1_* = 93 kcal mol^−1^) at pH 8.0. The additional flat, small peak centered around 47.5°C in the DSC curve was probably due to some deviation of CM denaturation from the two-state model in the proximity of pH 8.0.

**Table 3:** Dependence on pH of thermal transitions under the DSC envelope of Cpl-7.

pH	*T_m_*_1_(°C)	Δ*H*_1_ (kcal/mol)	*T_m_*_2_(°C)	Δ*H*_2_ (kcal/mol)	*T_m_*_3_(°C)	Δ*H*_3_ (kcal/mol)
6.0	49.3±0.4	54±3	55.8±0.1	109±3	64.0±0.2	57±3
6.7	48.9±0.4	71±6	54.8±0.2	121±10	63.2±0.2	75±4
7.0	50.4±0.2	39±4	52.1±0.1	98±1	63.5±0.5	59±2
7.8	47.5±0.5	27±10	46.4±0.1	82±3	61.5±0.2	53±2

### Stabilization of Cpl-7 by ligand binding; Identification of CW_7 targeted motifs in the bacterial cell wall

**Figure 7:**
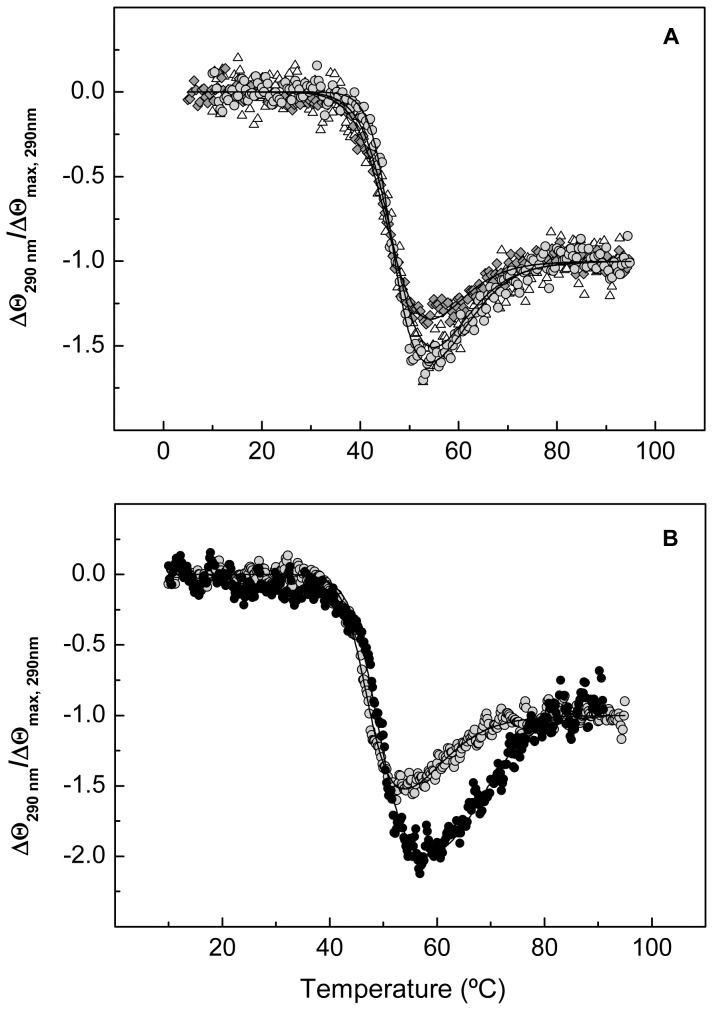
Influence of Cpl-7 potential ligands in the CM and CWBM structural stabilities. (**A**) shows the CD denaturation profiles of Cpl-7 at 290 nm in the absence (grey circles) and in the presence of 10 mM (GlcNAc)_6_ or 10 mM MurNAc-l-Ala-d-isoGln (white triangles and grey diamonds, respectively). (**B**) depicts the denaturation profiles in the absence and in the presence of 17.3 mM GlcNAc-MurNAc-l-Ala-d-isoGln (grey and black circles, respectively) at the same wavelength and pH.

The presence of CW_7-like repeats in a great variety of proteins putatively involved in the cell wall metabolism suggests the presence of a common type of cell wall-targeted motifs in all the encoding species, which include both Gram-positive and Gram-negative bacteria [14]. The identification of the modules involved in the different structural transitions of Cpl-7 made amenable the use of denaturation studies to search for the potential targets of the CW_7 repeats on the bacterial cell wall. To this aim, a series of experiments using different compounds structurally related to the peptidoglycan components (GlcNAc oligomers and small muropeptides) were carried out, monitoring the ellipticity variations at 290 nm during Cpl-7 denaturation. Data were collected at pH 8.0 where contributions from the CM and the CWBM are well resolved and where destabilization of the CM would not compromise the analysis of the results. Control experiments in the absence of tested ligands were run in parallel and all the assays were repeated at least twice. Addition of GlcNAc oligomers (GlcNAc_2_, GlcNAc_4_ and GlcNAc_6_) or MurNAc-l-Ala-d-isoGln at 10 mM final concentration did not affect Cpl-7 stability (Figure 7A). However, sample supplementation with GlcNAc-MurNAc-l-Ala-d-isoGln induced a concentration-dependent thermal up-shift of the last transition (Figure 7B; Table 4). This stabilization strongly points to GlcNAc-MurNAc-l-Ala-d-isoGln muropeptide as a component of the structural motif targeted by CW_7 repeats. The best fitting parameters derived both in the presence and in the absence of the assayed compounds are summarized in Table 4. A rough estimation of GlcNAc-MurNAc-l-Ala-d-isoGln affinity for the CWBM at its half-denaturation temperature was derived, using equation [2], from the thermal up-shift induced by the ligand binding. Assuming three identical binding sites (one per repeat) in the CWBM, the dissociation constant, *K_L,app_*, was estimated to be 12–15 mM, taking as 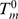 and Δ*H* in equation [2] the average of 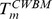 and 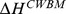 values derived from all the experiments performed with the unbound protein (59.3°C) and the GlcNAc-MurNAc-l-Ala-d-isoGln/Cpl-7 complex (53 kcal mol^−1^), respectively. Further extrapolation of current estimates to physiological temperatures would require an accurate determination of the enthalpy of ligand binding to CW_7 repeats as well as of its dependence with temperature, which is hardly affordable due to the ligand availability and binding affinity. The small increase (≈2°C) observed in the *T_m1_* upon addition of the disaccharide-dipeptide likely might reflect its recognition, though with much lower affinity, by the catalytic site. In this respect, binding of GlcNAc-MurNAc-l-Ala-d-isoGln to the active site of the Cpl-1 lysozyme has been demonstrated by crystallography [24].

**Table 4:** Influence of (GlcNAc)_n_ and muropeptides on the 290-nm CD denaturation profile of Cpl-7.

Compound added	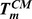	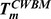	Δ*H^CM^*	Δ*H^CWBM^*	*f^CM^*	*f^CWBM^*
	(°C)	(°C)	(kcal/mol)	(kcal/mol)		
	46.8±0.4	60±1	85±5	41±3	1.83±0.05	0.83±0.05
10 mM (GlcNAc)_2_ 1	43.5±0.2	61±1	103±2	43±4	−1.7±0.1	0.7 ±0.1
10 mM (GlcNAc)_4_	47.4±0.4	57.1±0.2	61±1	51±10	1.95±0.25	0.95±0.25
10 mM (GlcNAc)_6_	48.4±0.1	58.9±0.3	73±2	57±6	−1.7±0.2	0.7±0.1
10 mM MurNAc-l-Ala-d-isoGln	45.6±0.4	58±2	60±3	44±6	−1.7±0.3	0.7±0.2
9 mM GlcNAc-MurNAc-l-Ala-d-isoGln^1^	43.7±0.1	66.2±0.3	89±3	56±4	−1.9±0.1	0.9±0.1
17.3 mM GlcNAc-MurNAc-l-Ala-d-isoGln	49.1±0.1	69±1	80±1	50±1	−2.0±0.1	1.0±0.1

Denaturation parameters were determined in 20 mM Pi buffer pH 8.0. *T_m_*, Δ*H* and *f* are defined as in Table 2; CM and CWBM superscripts indicate the module involved in the transition.

1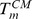 and 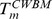 values in the absence of ligands for this batch of protein were 44.0°C and 60°C, respectively.

## Discussion

The potential of using purified recombinant endolysins in the prophylaxis and treatment of infections caused by many Gram-positive and a few Gram-negative pathogens has been highlighted during the past few years [2], [4], [6], [8], [25], [26]. As a new class of antimicrobials, the most relevant features of endolysins include a novel mode of antibacterial action, activity against antibiotic-resistant bacteria and a low probability of developing resistance. Besides, they usually have a narrow range of lytic activity that is often restricted to the host bacterial species infected by the phage. This stringent substrate specificity is frequently linked to the acquisition of cell wall-binding domains that specifically target certain structural motifs on the bacterial surface, which are distributed in genus-specific or even species/strain-specific manner [27]–[31]. Identification of motifs targeted by cell wall-binding domains is therefore one the most important aspects of endolysin research. The cell wall-binding motifs may also increase the enzyme-substrate proximity and facilitate the productive orientation of the hydrolyzable chain in the active site, and thereby the affinity and the lytic activity [10], [32]. Indeed, deletion of cell wall-binding motifs often results in substantial or complete abolishment of cell wall hydrolase activity [10], [16], [27], [33]–[35], a behavior that might be also related with the net charge of the catalytic domain/s [36].

In addition to adequate profiles of activity and specificity, the pharmacological or biotechnological use of endolysins requires a good structural stability. We have therefore characterized the conformational stability of the Cpl-7 endolysin and investigated the nature of the target recognized by the CW_7 repeats using CD and DSC. Cpl-7 unfolding closely reflects the endolysin modular structure, yet the complexity of the whole process indicates that the CM might not fold in just a single cooperative unit under most assayed conditions. By comparing the CD denaturation profiles of the full-length protein and of the isolated CM, transitions of the latter were clearly identified in Cpl-7 curves, which also allowed the assignment of those due to the CWBM denaturation. Both modules show a high conformational stability at neutral pH, with a maximum around pH 6.5, but their behaviours strongly differ in basic mediums, as the CM becomes extremely unstable above pH 8.0 and starts losing its native structure around 20°C at pH 8.5. According to CD and DSC data, CM denaturation proceeds in two steps that coalesce in a single process *T_m_*'s at around pH 8.0, only to separate again at higher values of pH. Such behavior reflects the high interdependence of the CM regions accounting for both transitions [20], [37]. In contrast, the ellipticity changes assigned to the CWBM slightly vary with pH and the whole module tends to unfold as a single cooperative unit. Although this denaturation model may represent a simplification of the real process, it provides a good description of Cpl-7 thermal unfolding with a relatively simple analysis of the experimental data.

The low influence of the pH on the CWBM stability agrees well with the absence in CW_7 repeats of titratable side-chains with pK_a_ values in the range of pH assayed. Variations in its net charge are essentially due to completion of carboxylic group ionization (≈−0.24 charges) and the beginning of tyrosine and lysine deprotonation (≈−0.39 charges). The presence of one additional step below pH 7.0 might reflect the establishment of some interactions with either the linker or the CM that would be lost as the net negative charge of the three structural elements (CM, linker and CWBM) of Cpl-7 increase, mainly due, again, to completion of carboxylic group ionization (estimated net charges at neutral pH for the CM, the linker, and the CWBM would be around −10.9, −4.0, and −14.9, respectively).

The strong drop in the *T_m_* of the CM transition associated to the loss of the 290-nm positive band could be due to deprotonation of the N-terminal amino group (intrinsic pK_a_≈7.7) and/or to histidines stabilized by interactions with neighboring groups. The histidines of the CM, located at positions 14 (loop between *β*1 and *α*1), 60 (*β*3) and 96 (loop between *β*4 and *α*4), are all of them highly apart from the β-barrel side devoid of α-helices (*β*6 to *β*8), which is likely involved in this transition (Figure S3). By the contrary, the N-terminal amino group is close to W173 (*β*7), which is about 3 Å away from W149 (*β*6). On the other hand, the CM transition accounting for ellipticity variations at 195 nm (a maximum in the α-helix CD spectrum) shows a moderate dependence on pH, and the stability decrease observed above pH 7.0 might be due, again, to variations in the protonation state of histidines (all of them in the barrel side containing the α-helices) and/or the N-terminal amino group, though the influence of protonation/deprotonation equilibriums is lower than for the other CM transition.

As already mentioned, the CW_7 motifs have been identified in a great number of putative proteins encoded by both Gram-positive and Gram-negative bacteria and also by phages infecting Gram-positive bacteria [14]. Gram-negative bacteria lack secondary glycopolymers commonly attached to the peptidoglycan or to the membrane lipids in Gram-positive microorganisms and their lipopolysaccharides are located at the outermost part of the outer membrane [38]. These facts, and the observation that most CW_7 carrying enzymes seems to be involved in cell wall metabolism, strongly suggest that CW_7 motifs would target a common structure of the peptidoglycan network, shared by both Gram-negative and Gram-positive microorganisms. This notion is also supported by the great differences existing in the compositions of (lipo)teichoic acids of *S. pneumoniae* (ribitol-phosphate pentasaccharide polymers) [39], *Streptococcus pyogenes* or *S. agalactiae* (glycerol-phosphate polymers) [40]. These three streptococci are infected by bacteriophages encoding CW_7 containing endolysins [14] and their envelopes lack other secondary glycopolymers. On the other hand, the D-Ala moieties covalently attached to either glycerol- or ribitol-phosphate residues of their respective (lipo)teichoic acids are absent in the pneumococcal strain R6 [41], a laboratory non-encapsulated strain susceptible to the Cpl-7 endolysin [13]. Indeed, our denaturation studies in the presence of peptidoglycan analogues strongly indicated that recognition of the bacterial surface by CW_7 motifs implies its direct binding to the cell wall muropeptide. As shown in Table 4, at comparable ligand concentrations, only the GlcNAc-MurNAc-l-Ala-d-isoGln muropeptide stabilizes the CWBM, evidencing that both the glycan chain and the stem peptide are relevant for CW_7/peptidoglycan interactions. Moreover, the failure of the monosaccharide-dipeptide and GlcNAc oligosaccharides in promoting the stabilization of the CWBM highlights the relevance of the GlcNAc ring, the lactyl-group of MurNAc and/or the stem peptide for cell wall targeting by the CW_7 motifs. Nevertheless, the differences observed between the denaturation profiles obtained in the absence and in the presence of MurNAc-l-Ala-d-isoGln above 50°C might reflect a rather loose interaction with the monosaccharide-dipeptide (Figure 7A). Whether CW_7/muropeptide interactions expand to other residues of the stem peptide or to the interpeptide bridges have yet to be elucidated. Although the more abundant peptide bridges in pneumococci differ from those in other streptococci [42], sequence variation of the CW_7 motifs might accommodate this variability and improve substrate specificity, as seems to happen in *LysM* domains (Pfam PF01476) that have evolved to target different types of peptidoglycans in bacteria, and chitin-like compounds in eukaryotes or viral glycoproteins [42]–[44].

The apparent dissociation constant of GlcNAc-MurNAc-l-Ala-d-isoGln from CW_7 repeats at denaturation temperatures is in the high millimolar range, and currently available information does not allow its extrapolation to physiological temperatures. However, taking into account that the ratio between GlcNAc-MurNAc-l-Ala-d-isoGln *α*- and *β*-anomers in solution is 70:30, the value of *K_L,app_* might be up-to 3.3 times the intrinsic dissociation constant, *K_L_*, if the CWBM preferentially binds to the *β*-anomer (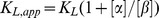), which is the form present in the cell wall. Such an effect could reduce the dissociation constant for the *β*-anomer down to 4–5 mM, which is about twice the value estimated at 25°C for choline dissociation from the Cpl-1 endolysin [45].

One of the major advantages of multivalent interactions is their potential to achieve effective adhesion between binding partners, even when individual, monovalent interactions are weak [46], as seems to occur with association of GlcNAc-MurNAc-l-Ala-d-isoGln to CW_7 repeats *in vitro*. Therefore, the strength and selectivity of Cpl-7 binding *in vivo* probably relies on the ability of its CWBM to create multiple, weak, noncovalent bonds with peptidoglycan muropeptides. Indeed, a highly efficient endolysin would require an easy transit across the polymeric substrate to reach new cleavable bonds, which would be impaired by a strong binding and a slow dissociation rate. On the contrary, creation of multiple weak contacts will facilitate Cpl-7 diffusion by means of a binding/release mechanism without completely detaching from the cell wall, as observed in other multivalent systems [47], [48]. At the same time, the effective binding constant would be higher than the association constants for each individual interaction. One important factor for the affinity increase is the entropic cost of the loss of translational and rotational degrees of freedom inherent to complex formation. If the CWBM of Cpl-7 simultaneously interacts with more than one muropeptide moiety of the peptidoglycan scaffold, the loss of translational and rotational entropy would only occur at the first binding event, since subsequent binding steps are monomolecular processes. This means reducing such unfavorable entropic term to a third of the total penalty paid in Cpl-7 association to three monovalent ligands, with the subsequent increase of binding affinity. Nevertheless, the extent to which the affinity constant is actually enhanced depends also on both the structure and the geometry of the protein receptor and the precise arrangement of the ligands. In this respect, the size of each CW_7 repeat seems large enough to assume that it can accommodate a GlcNAc-MurNAc-l-Ala-d-isoGln moiety without interfering with the association of other muropeptide moieties to its contiguous repeats [14], [49].

Modular recombination of proteins is one of the driving forces in evolution and permits a rapid adaptation to new environmental conditions [50]–[52]. This phenomenon has been observed for many cell wall- (i.e., polymeric-) degrading substrates [53]–[55] and confirmed by the construction of functional chimeric lysins [13], [36], [56]–[59]. Whether the number and type of acquired motifs is optimized or not for a given protein is still unknown. Apparently, small differences in affinity for isolated, monovalent peptidoglycan ligands might be amplified by multiple CWBM-peptidoglycan scaffold interactions to achieve the required substrate specificity, even at species or strain level. Consistent with this idea, CW_7 motifs appear frequently associated to LysM motifs in lysozymes [14], and the acquisition of one or two CW_7-like motifs by the endopeptidase module of the λSa2 endolysin not only increases the activity on several streptococcal strains, but it was shown to be essential for its activity on staphylococci [16]. Combination of several cell wall-binding motifs in a single polypeptide chain may, therefore, facilitate rapid evolution to new specificities while using existing cell-wall surface receptors. In this regard, our finding that the CM and the CWBM in Cpl-7 act as essentially independent folding units gives support to the possibility of readily combining both modules with other catalytic modules and/or cell wall-binding motifs to yield tailored chimeric lysins with different/broader spectra of lytic specificities or higher bactericidal activities. Besides, although the three CW_7 repeats of Cpl-7 unfold basically as a single cooperative unit, the presence of just one or two motifs in other proteins indicates that a single CW_7 motif can probably form a structurally stable domain.

## Supporting Information

Figure S1 **Dependence of Cpl-7 specific activity on pH.** Measurements were performed at 37°C in Pi buffer, pH 7.0, using [*methyl*-^3^H]choline-labeled pneumococcal cell walls as substrate.
(PDF)

Figure S2 **Influence of pH on the dependence of Cpl-7 near-UV spectra with temperature.**
(PDF)

Figure S3 **Distribution of tryptophans and histidines in the 3D structure of the CM of Cpl-7.** Side-chains of selected residues in stick representation.
(PDF)

## References

[1] Fischetti VA (2005) Bacteriophage lytic enzymes: novel anti-infectives. Trends Microbiol 13: 491–496. 16125935 10.1016/j.tim.2005.08.007

[2] Borysowski J, Górski A. (2010) Anti-staphylococcal lytic enzymes. In Villa TG, Veiga-Crespo P, editors. Enzybiotics: Antibiotic Enzymes as Drugs and Therapeutics, John Wiley & Sons, Inc., Hoboken, NJ, USA. pp. 149–172.

[3] Loeffler JM, Nelson D, Fischetti VA (2001) Rapid killing of *Streptococcus pneumoniae* with a bacteriophage cell wall hydrolase. Science 294: 2170–2172. 11739958 10.1126/science.1066869

[4] Borysowski J, Weber-Dabrowska B, Górski A (2006) Bacteriophage endolysins as a novel class of antibacterial agents. Exp Biol Med 231: 366–377. 10.1177/153537020623100402 16565432

[5] Schuch R, Nelson D, Fischetti VA (2002) A bacteriolytic agent that detects and kills *Bacillus anthracis* . Nature 418: 884–889. 12192412 10.1038/nature01026

[6] Hermoso JA, García JL, García P (2007) Taking aim on bacterial pathogens: from phage therapy to enzybiotics. Curr Opin Microbiol 10: 461–472. 17904412 10.1016/j.mib.2007.08.002

[7] Fenton M, Ross P, McAuliffe O, O'Mahony J, Coffey A (2010) Recombinant bacteriophage lysins as antibacterials. Bioeng Bugs 1: 9–16. 21327123 10.4161/bbug.1.1.9818 PMC3035150

[8] Callewaert L, Walmagh M, Michiels CW, Lavigne R (2011) Food applications of bacterial cell wall hydrolases. Curr Opin Biotechnol 22: 164–171. 21093250 10.1016/j.copbio.2010.10.012

[9] García P, García JL, García E, Sánchez-Puelles JM, López R (1990) Modular organization of the lytic enzymes of *Streptococcus pneumoniae* and its bacteriophages. Gene 86: 81–88. 2311937 10.1016/0378-1119(90)90116-9

[10] Sanz JM, Díaz E, García JL (1992) Studies on the structure and function of the *N*-terminal domain of the pneumococcal murein hydrolases. Mol Microbiol 6: 921–931. 1351240 10.1111/j.1365-2958.1992.tb01542.x

[11] García P, García JL, López R, García E (2005) Pneumococcal Phages: In Waldor M K, Friedman DLI, Adhya S, editors. Phages: Their Role in Bacterial Pathogenesis and Biotechnology, ASM Press, Washington, DC. pp. 335–361.

[12] López R, García E, García P, García JL (2004) Cell Wall Hydrolases. In Tuomanen EI, Mitchell TJ, Morrison DA, Spratt BG, editors. The Pneumococcus, ASM Press, Washington, DC. pp. 75–88.

[13] Diaz E, López R, Garcia JL (1991) Chimeric pneumococcal cell wall lytic enzymes reveal important physiological and evolutionary traits. J Biol Chem 266: 5464–5471. 1672313

[14] Bustamante N, Campillo NE, García E, Gallego C, Pera B, et al (2010) Cpl-7, a lysozyme encoded by a pneumococcal bacteriophage with a novel cell wall-binding motif. J Biol Chem 285: 33184–33196. 20720016 10.1074/jbc.M110.154559 PMC2963342

[15] Hermoso JA, Monterroso B, Albert A, Galán B, Ahrazem O, et al (2003) Structural basis for selective recognition of pneumococcal cell wall by modular endolysin from phage Cp-1. Structure 11: 1239–1249. 14527392 10.1016/j.str.2003.09.005

[16] Donovan DM, Foster-Frey J (2008) LambdaSa2 prophage endolysin requires Cpl-7-binding domains and amidase-5 domain for antimicrobial lysis of streptococci. FEMS Microbiol Lett 287: 22–33. 18673393 10.1111/j.1574-6968.2008.01287.x

[17] Laue TM, Shah BD, Ridgeway TM, Pelletier SL (1992) Computer-aided interpretation of analytical sedimentation data for proteins. In Harding SE, Rowe AJ, Horton JC, editors. Analytical Ultracentrifugation in Biochemistry and Polymer Science. Royal Soc Chem, Cambridge, UK. pp. 90–124,

[18] Moreno FJ, Quintanilla-López JE, Lebrón-Aguilar R, Olano A, Sanz ML (2008) Mass spectrometric characterization of glycated β-lactoglobulin peptides derived from galacto-oligosaccharides surviving the *in vitro* gastrointestinal digestion. J Am Soc Mass Spectrom 19: 927–937. 18467121 10.1016/j.jasms.2008.04.016

[19] Carreira A, Menéndez M, Reguera J, Almendral JM, Mateu MG (2004) In vitro assembly of a parvovirus capsid and effect on capsid stability of heterologous peptide insertions in surface loops. J Biol Chem 279: 6517–6525. 14660623 10.1074/jbc.M307662200

[20] Brandts JF, Hu CQ, Lin LN, Mas MT (1989) A simple model for proteins with interacting domains. Applications to scanning calorimetry data. Biochemistry 28: 8588–8596. 2690944 10.1021/bi00447a048

[21] Strickland EH (1974) Aromatic contributions to circular dichroism spectra of proteins. CRC Crit Rev Biochem 2: 113–175. 4591332 10.3109/10409237409105445

[22] Sanchez-Ruiz JM (1992) Theoretical analysis of Lumry-Eyring models in differential scanning calorimetry. Biophys J 61: 921–935. 19431826 10.1016/S0006-3495(92)81899-4 PMC1260351

[23] Sanz J, García JL, Laynez J, Usobiaga P, Menéndez M (1993) Thermal stability and cooperative domains of CPL-1 lysozyme and its NH2- and COOH-terminal modules. J Biol Chem 268: 6125–6130. 8454587

[24] Pérez-Dorado I, Campillo NE, Monterroso B, Hesek D, Lee M, et al (2007) Elucidation of the molecular recognition of bacterial cell wall by modular pneumococcal phage endolysin CPL-1. J Biol Ch*e*m 282: 24990–24999. 17581815 10.1074/jbc.M704317200

[25] Loessner MJ (2005) Bacteriophage endolysins – current state of research and applications. Curr Opin Microbiol 8: 480–487. 15979390 10.1016/j.mib.2005.06.002

[26] Fischetti VA (2010) Bacteriophage endolysins: A novel anti-infective to control Gram-positive pathogens. Int J Med Microbiol 300: 357–362. 20452280 10.1016/j.ijmm.2010.04.002 PMC3666336

[27] Loessner MJ, Kramer K, Ebel F, Scherer S (2002) C-terminal domains of *Listeria monocytogenes* bacteriophage murein hydrolases determine specific recognition and high-affinity binding to bacterial cell wall carbohydrates. Mol Microbiol 44: 335–349. 11972774 10.1046/j.1365-2958.2002.02889.x

[28] Farkašovská J, Godány A, Vlcek C (2003) Identification and characterization of an endolysin encoded by the *Streptomyces aureofaciens* phage mu 1/6. Folia Microbiol (Praha) 48: 737–744. 15058185 10.1007/BF02931507

[29] López R, García E (2004) Recent trends on the molecular biology of pneumococcal capsules, lytic enzymes, and bacteriophage. FEMS Microbiol Rev 28: 553–580. 15539074 10.1016/j.femsre.2004.05.002

[30] Briers Y, Volckaert G, Cornelissen A, Lagaert S, Michiels CW, et al (2007) Muralytic activity and modular structure of the endolysins of *Pseudomonas aeruginosa* bacteriophages φKZ and EL. Mol Microbiol 65: 1334–1344. 17697255 10.1111/j.1365-2958.2007.05870.x

[31] Vollmer W, Joris B, Charlier P, Foster S (2008) Bacterial peptidoglycan (murein) hydrolases. FEMS Microbiol Rev 32: 259–286. 18266855 10.1111/j.1574-6976.2007.00099.x

[32] Hermoso JA, Lagartera L, González A, Stelter M, García P, et al (2005) Insights into pneumococcal pathogenesis from the crystal structure of the modular teichoic acid phosphorylcholine esterase Pce. Nat Struct Mol Biol 12: 533–538. 15895092 10.1038/nsmb940

[33] Sánchez-Puelles JM, García JL, López R, García E (1987) 3′-End modifications of the *Streptococcus pneumoniae lytA* gene: role of the carboxy terminus of the pneumococcal autolysin in the presence of enzymatic activation (conversion). Gene 61: 13–19. 2895040 10.1016/0378-1119(87)90360-x

[34] García P, González MP, García E, García JL, López R (1999) The molecular characterization of the first autolytic lysozyme of *Streptococcus pneumoniae* reveals evolutionary mobile domains. Mol Microbiol 33: 128–138. 10411730 10.1046/j.1365-2958.1999.01455.x

[35] Porter CJ, Schuch R, Pelzek AJ, Buckle AM, McGowan S, et al (2007) The 1.6 Å crystal structure of the catalytic domain of PlyB, a bacteriophage lysin active against *Bacillus anthracis* . J Mol Biol 366: 540–550. 17182056 10.1016/j.jmb.2006.11.056

[36] Low LY, Yang C, Perego M, Osterman A, Liddington R (2011) The role of net charge on the catalytic domain and the influence of the cell-wall binding domain on the bactericidal activity, specificity and host-range of phage lysins. J Biol Chem 286: 34391–34403. 21816821 10.1074/jbc.M111.244160 PMC3190764

[37] Luque I, Leavitt SA, Freire E (2002) The linkage between protein folding and functional cooperativity: two sides of the same coin?. Annu Rev Biophys Biomol Struct 31: 235–256. 11988469 10.1146/annurev.biophys.31.082901.134215

[38] Weidenmaier C, Peschel A (2008) Teichoic acids and related cell-wall glycopolymers in Gram-positive physiology and host interactions. Nat Rev Microbiol 6: 276–287. 18327271 10.1038/nrmicro1861

[39] Seo HS, Cartee RT, Pritchard DG, Nahm MH (2008) A new model of pneumococcal lipoteichoic acid structure resolves biochemical, biosynthetic, and serologic inconsistencies of the current model. J Bacteriol 190: 2379–2387. 18245291 10.1128/JB.01795-07 PMC2293179

[40] Neuhaus FC, Baddiley J (2003) A continuum of anionic charge: structures and functions of D-alanyl-teichoic acids in gram-positive bacteria. Microbiol Mol Biol Rev 67: 686–723. 14665680 10.1128/MMBR.67.4.686-723.2003 PMC309049

[41] Draing C, Pfitzenmaier M, Zummo S, Mancuso G, Geyer A, et al (2006) Comparison of lipoteichoic acid from different serotypes of *Streptococcus pneumoniae* . J Biol Chem 281: 33849–33859. 16943191 10.1074/jbc.M602676200

[42] Scott JR, Barnett TC (2006) Surface proteins of gram-positive bacteria and how they get there. Annu Rev Microbiol 60: 397–423. 16753030 10.1146/annurev.micro.60.080805.142256

[43] Buist G, Steen A, Kok J, Kuipers OP (2008) LysM, a widely distributed protein motif for binding to (peptido)glycans. Mol Microbiol 68: 838–847. 18430080 10.1111/j.1365-2958.2008.06211.x

[44] Ohnuma T, Onaga S, Murata K, Taira T, Katoh E (2008) LysM domains from *Pteris ryukyuensis* chitinase-A. A stability study and characterization of the chitin-binding site. J Biol Chem 283: 5178–5187. 18083709 10.1074/jbc.M707156200

[45] Monterroso B, Sáiz JL, García P, García JL, Menéndez M (2008) Insights in structure and function relationships in pneumococcal lysozymes cell-wall lysozymes: LytC and Cpl-1. J Biol Chem 283: 28618–28628. 18667432 10.1074/jbc.M802808200 PMC2661411

[46] Mammen M, Choi S-K, Whitesides GM (1998) Polyvalent interactions in biological systems: Implications for design and use of multivalent ligands and inhibitors. Angew Chem Int Ed 37: 2755–2794. 10.1002/(SICI)1521-3773(19981102)37:20<2754::AID-ANIE2754>3.0.CO;2-3 29711117

[47] Dam TK, Brewer CF (2008) Effect of clustered epitopes in multivalent ligand-receptor interactions. Biochemistry 47: 8470–8476. 18652478 10.1021/bi801208b

[48] Perl A, Gómez-Casado A, Thompson D, Dam HH, Honkheijn P, et al (2011) Gradient-driven motions of multivalent ligand molecules along a surface functionalized with multiple receptors. Nat Chem 3: 317–322. 21430692 10.1038/nchem.1005

[49] Meroueh SO, Bencze KZ, Hesek D, Mijoon L, Fisher JF, et al (2006) Three-dimensional structure of the bacterial cell wall peptidoglycan. Proc Natl Acad Sci USA 103: 4404–4409. 16537437 10.1073/pnas.0510182103 PMC1450184

[50] Visweswaran GRR, Dijkstra BW, Kok J (2011) Murein and pseudomurein cell wall binding domains of bacteria and archaea–a comparative view. Appl Microbiol Biotechnol 92: 921–928. 22012341 10.1007/s00253-011-3637-0 PMC3210951

[51] Trifonov EN, Frenkel ZM (2009) Evolution of protein modularity. Curr Opin Struct Biol 19: 335–340. 19386484 10.1016/j.sbi.2009.03.007

[52] Bornberg-Bauer E, Huylmans A-K, Sikosek T (2010) How do new proteins arise?. Curr Opin Struct Biol 20: 390–396. 20347587 10.1016/j.sbi.2010.02.005

[53] García E, García JL, García P, Arrarás A, Sánchez-Puelles JM, et al (1988) Molecular evolution of lytic enzymes of *Streptococcus pneumoniae* and its bacteriophages. Proc Natl Acad Sci USA 85: 914–918. 3422470 10.1073/pnas.85.3.914 PMC279667

[54] Croux C, Ronda C, López R, García JL (1993) Role of the C-terminal domain of the lysozyme of *Clostridium acetobutylicum* ATCC 824 in a chimeric pneumococcal-clostridial cell wall lytic enzyme. FEBS Lett 336: 111–114. 7903254 10.1016/0014-5793(93)81621-6

[55] Sheehan MM, García JL, López R, García P (1997) The lytic enzyme of the pneumococcal phage Dp-1: a chimeric lysin of intergeneric origin. Mol Microbiol 25: 717–725. 9379901 10.1046/j.1365-2958.1997.5101880.x

[56] Díaz E, López R, García JL (1990) Chimeric phage-bacterial enzymes: a clue to the modular evolution of genes. Proc Natl Acad Sci USA 87: 8125–8129. 1978320 10.1073/pnas.87.20.8125 PMC54905

[57] Croux C, Ronda C, López R, García JL (1993) Interchange of functional domains switches enzyme specificity: construction of a chimeric pneumococcal-clostridial cell wall lytic enzyme. Mol Microbiol 9: 1019–1025. 7934908 10.1111/j.1365-2958.1993.tb01231.x

[58] Manoharadas S, Witte A, Bläsi U (2009) Antimicrobial activity of a chimeric enzybiotic towards *Staphylococcus aureus* . J Biotechnol 139: 118–123. 18940209 10.1016/j.jbiotec.2008.09.003

[59] Visweswaran G, Dijkstra B, Kok J (2012) A genetically engineered protein domain binding to bacterial murein, archaeal pseudomurein, and fungal chitin cell wall material. Appl Microbiol Biotechnol doi:10.1007/s00253-00012-03871-00250. 10.1007/s00253-012-3871-0 PMC3466432 22262228

