# Supplementary figures and images for: Thermal Stability of Cpl-7 Endolysin from the Streptococcus pneumoniae Bacteriophage Cp-7; Cell Wall-Targeting of Its CW_7 Motifs
Source: PLoS One. 2012 Oct 8;7(10):e46654. doi: 10.1371/journal.pone.0046654 (PMC3466307; doi:10.1371/journal.pone.0046654)

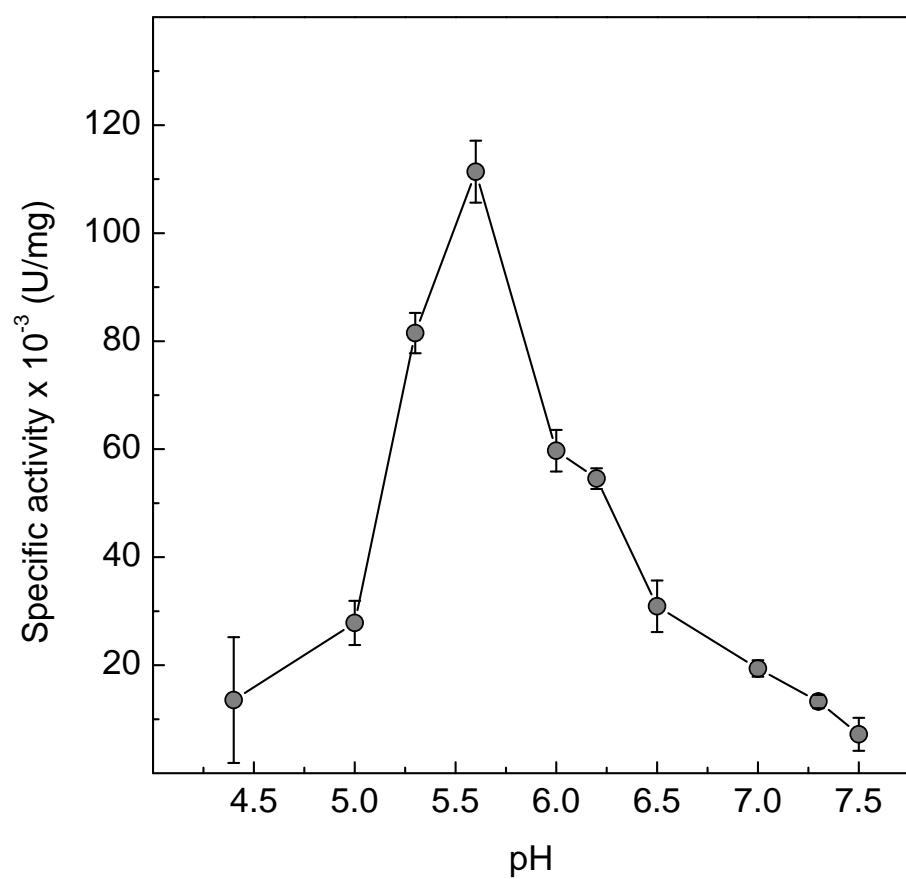

Supplementary Fig.S1

Supplement: Figure S1 — Dependence of Cpl-7 specific activity on pH. Measurements were performed at 37°C in Pi buffer, pH 7.0, using [methyl-3H]choline-labeled pneumococcal cell walls as substrate. (PDF) [file pone.0046654.s001.pdf]

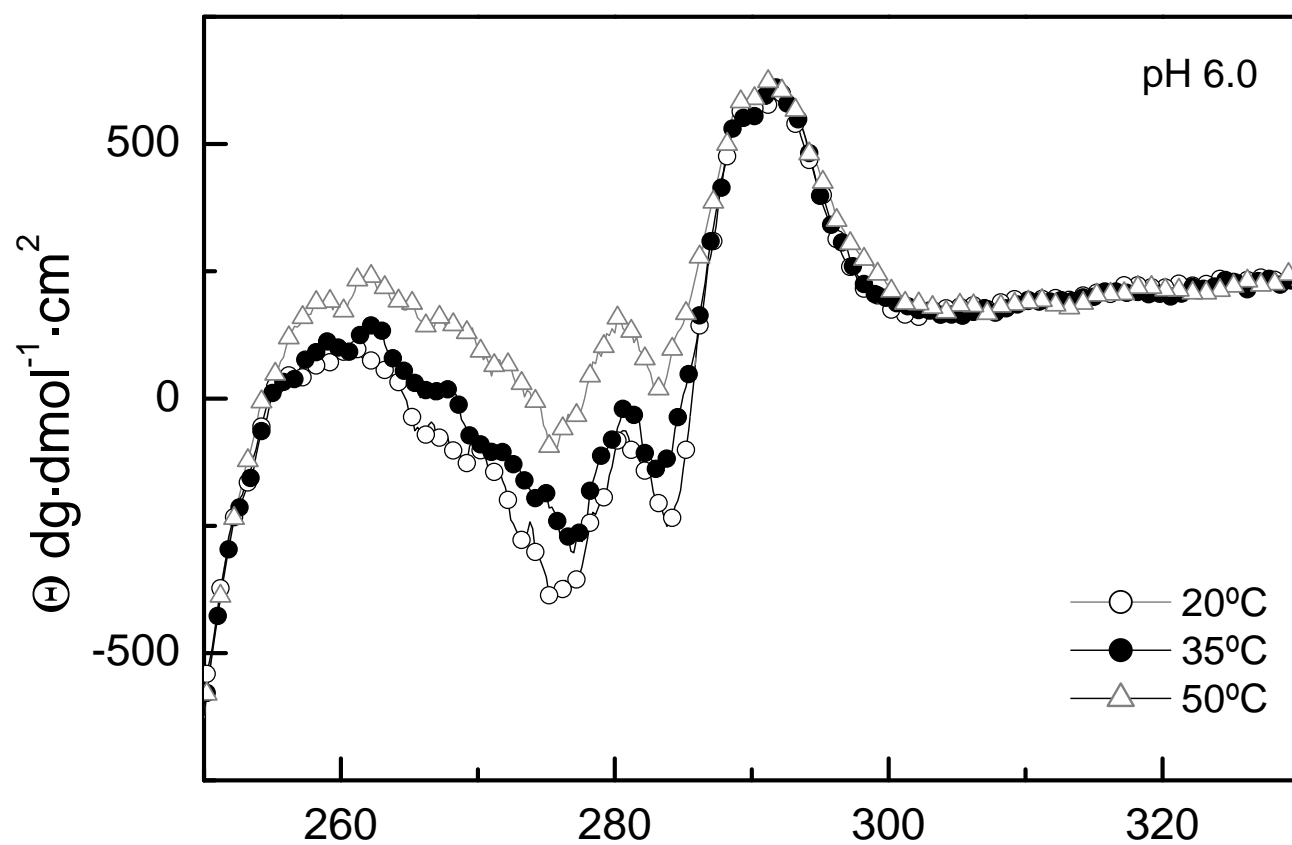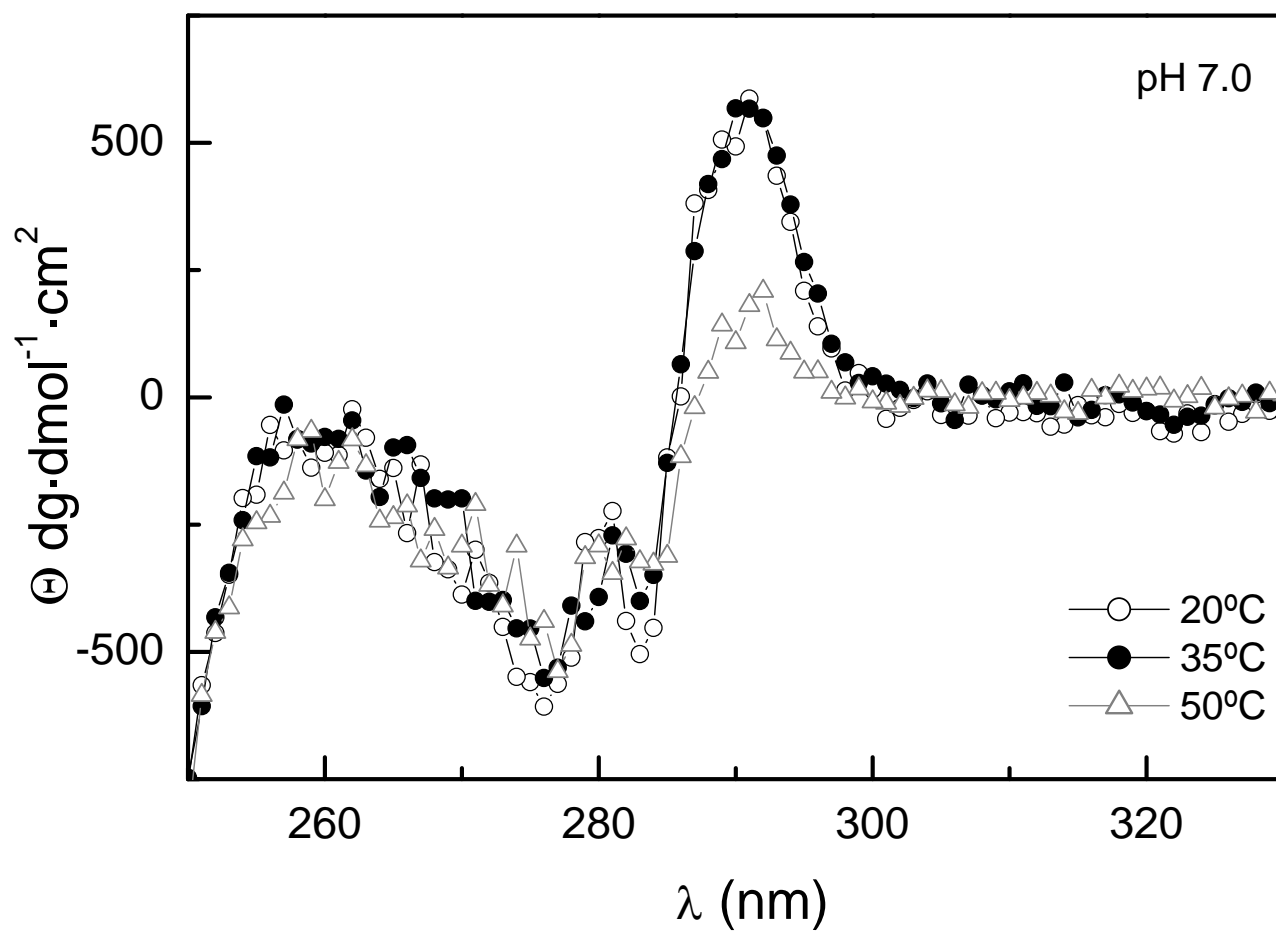

Supplementary Fig. S2

Supplement: Figure S2 — Influence of pH on the dependence of Cpl-7 near-UV spectra with temperature. (PDF) [file pone.0046654.s002.pdf]

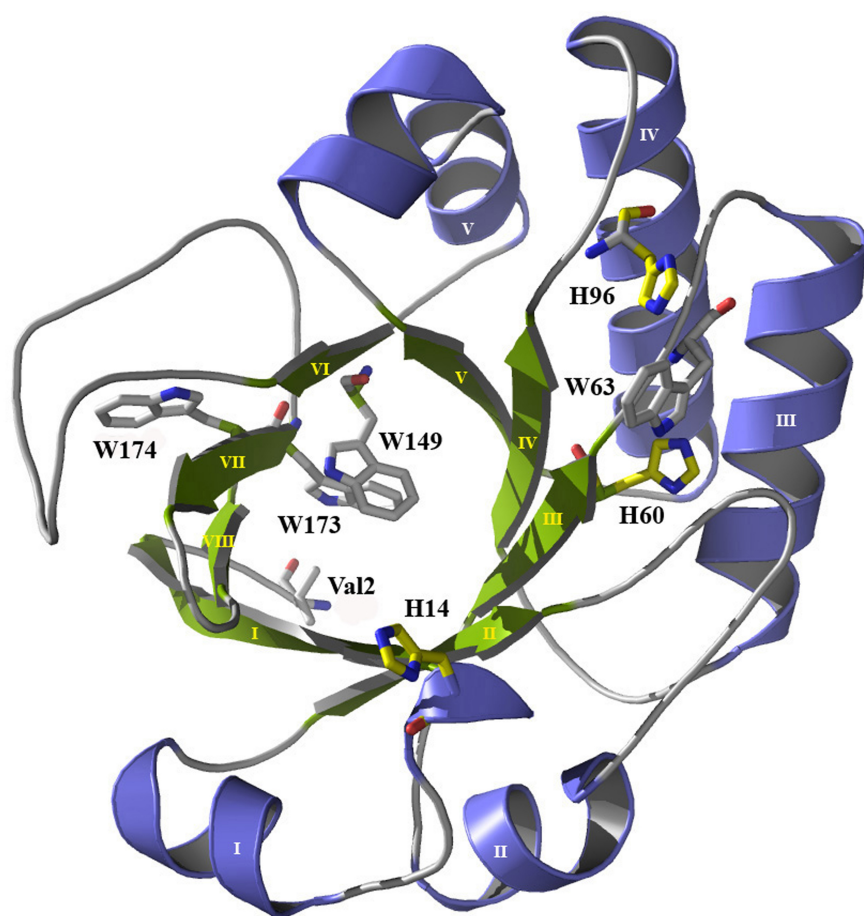

Supplementary Fig. S3

Supplement: Figure S3 — Distribution of tryptophans and histidines in the 3D structure of the CM of Cpl-7. Side-chains of selected residues in stick representation. (PDF) [file pone.0046654.s003.pdf]
